# Cellular Effects of *Enterobacteriaceae* Polysaccharide Colanic Acid

**DOI:** 10.3390/ijms25158017

**Published:** 2024-07-23

**Authors:** Sofia A. Tsvetikova, Alina A. Zabavkina, Olesia Ivankova, Elena I. Koshel

**Affiliations:** SCAMT Laboratory, National Research University ITMO, St. Petersburg 197101, Russia; zabavkina@scamt-itmo.ru (A.A.Z.); ivankova@scamt-itmo.ru (O.I.)

**Keywords:** colanic acid, exopolysaccharide, *Escherichia coli*, cytotoxicity, mitochondria, antioxidants

## Abstract

Colanic acid (CA) is an exopolysaccharide found in *Enterobacteriaceae*. Recently, its ability to stimulate physical activity in mice and to prolong the lifespan of invertebrates has been described. In the current work, we use standard MTT assay, fluorescence microscopy, and flow cytometry to describe CA action on several cell lines of different origins. We observed slight antiproliferative activity against colorectal cancer (HCT-116), neuroblastoma (IMR-32), and myoblast (C2C12) cell lines at a concentration of 256 μg/mL, while other cell lines of non-cancerous origin (Vero, HPF) did not show any decrease in the MTT assay. In all cell lines, we observed a rearrangement of mitochondria localization using fluorescence microscopy. CA induces cell differentiation in the myoblast cell line (C2C12) at concentrations of 50–200 μg/mL. Briefly, we observed that the number of apoptotic cells increased and the metabolic activity in the MTT assay decreased, which was accompanied by changes in cell morphology, the quantity of ROS, and the potential of the mitochondrial membrane. Taken together, these results indicate that CA is specific in cytotoxicity to cell lines of different origins and can impact mitochondria and differentiation, consistent with its potential geroprotective function.

## 1. Introduction

Bacterial polysaccharides can be produced outside the cell as a component of lipopolysaccharide [1], capsule [2], and biofilm matrix [3]. Most bacterial polysaccharides are recognized by host cells with pattern recognition receptors (PRRs), mainly the mannose receptor (CD206) [4] and Toll-like receptors, especially TLR2 [5] and TLR4 [6]. Through these receptors, bacterial polysaccharides, such as polysaccharide A of *Bacteroides fragilis* [7], can exert an immunomodulatory function in the host organism [7,8,9]. Other bacterial polysaccharides can have antioxidant properties [10] and the ability to suppress proliferation [11,12].

Colanic acid (CA) is an extracellular polysaccharide commonly produced by several species of the *Enterobacteriaceae* family, including *Escherichia coli* [13,14,15]. CA is a branched, fucose-rich heteropolysaccharide [16]. CA protects the cell from environmental conditions as a component of the bacterial capsule and as a component of the biofilm matrix [17,18]. Recent studies have shown that CA can be produced in nematodes and fruit flies by introduced members of the gut microbiota [19,20], resulting in increased longevity and protection against age-related conditions in the host. Several studies indicate that CA is immunogenic in mice when administered by injection [13,14,21], while data on oral administration in water solution show low toxicity in cell lines and animals and the ability to stimulate physical activity [22]. The mechanism of action of CA is related to mitochondrial dynamics and the mitochondrial unfolded protein response (UPRmt) [19]. Therefore, further investigation into the mechanism of action and targets of CA is required to evaluate its therapeutic and biotechnological potential.

In the current work, we aimed to study CA in five cell lines of different origins: HCT-116, IMR-32, HPF, Vero, and C2C12. We evaluated the ability of CA to affect survival, cell and mitochondria morphology, and C2C12 differentiation. The obtained results can be considered for the further development of CA-based therapies.

## 2. Results

### 2.1. CA Cytotoxicity

**Figure 1:**
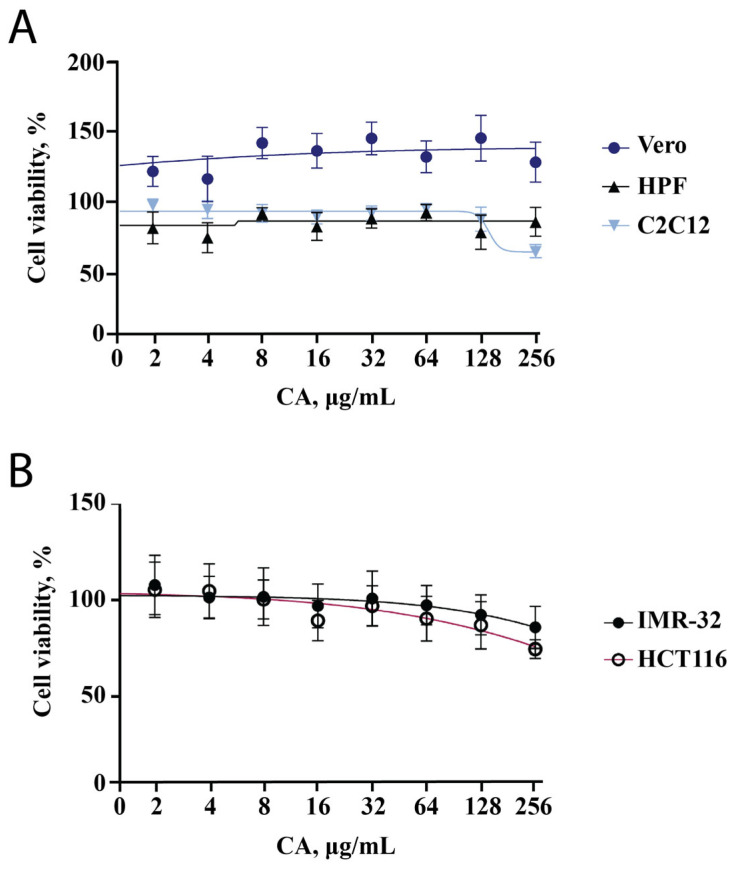
CA influence on metabolic activity of five different cell lines. Points are the mean of two independent experiments (each conducted in triplicate); error bars indicate standard deviation. (**A**) MTT assay results for Vero, HPF, C2C12 cell lines. (**B**) MTT assay results for cell lines of cancerous origin (IMR-32, HCT116).

We decided to start the evaluation of the effect of CA on cell lines with the MTT assay since this test reflects changes in cell metabolic activity and cytotoxicity of the tested substance. We used two cell lines of cancer origin (HCT-116 and IMR-32) and three other cell lines (HPF, C2C12, Vero). Two non-cancerous cell lines (Vero and HPF) showed no decrease in metabolic activity across a wide range of concentrations (2–256 μg/mL, Figure 1A). The metabolic activity of IMR-32 and HCT-116 was found to decrease with increasing CA concentration; however, it remained higher than 80% (Figure 1B). This indicates that CA exhibits a slight antiproliferative activity against tumor cells, which has been previously described in nematodes [19] and shown for other bacterial polysaccharides [23,24,25]. At a CA concentration of 256 μg/mL, the metabolic activity of the C2C12 cell line was reduced to 70% (Figure 1A). This result can be related to both toxicity and differentiation, which was analyzed in the subsequent experiments.

### 2.2. Cell Morphology Analysis

**Figure 2:**
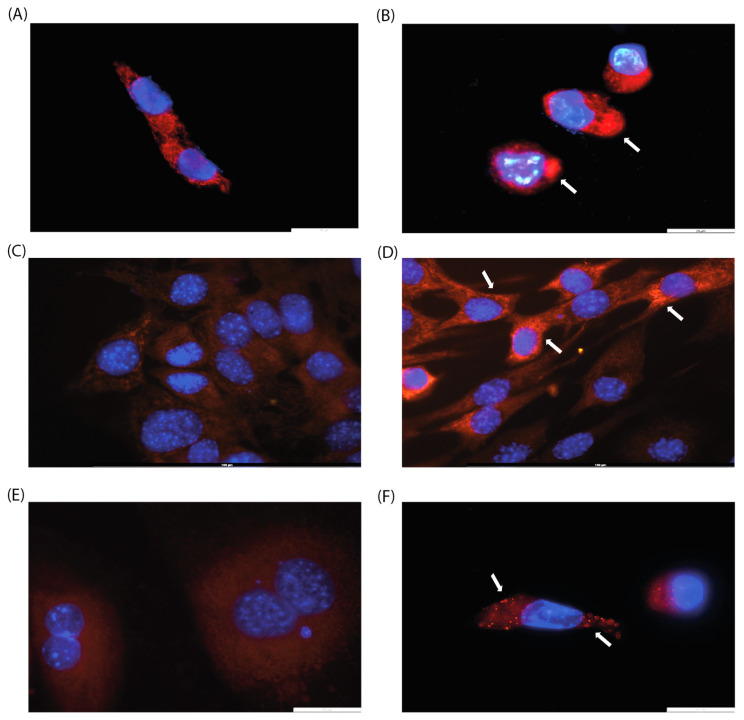
Evaluation of mitochondrial morphology of (**A**,**B**) HCT-116, (**C**,**D**) C2C12, and (**E**,**F**) IMR-32 cell lines after 24 h of incubation with CA (200 µg/mL, (**B**,**D**,**F**)) or without it (control, (**A**,**C**,**E**)). Arrows indicate mitochondrial clusters. Magnification: ×100.

Since the effect of CA on mitochondrial arrangement has been previously described [19], we decided to perform cell microscopy. We analyzed cell morphology by DAPI and MitoTracker staining to visualize mitochondrial localization since previous studies have shown that CA induces mitochondrial fission [19]. Three cell lines were selected for this purpose: HPF, which showed no change in metabolic activity in MTT assay, and HCT-116 and C2C12, which exhibited a moderate decrease in metabolic activity. In all cell lines examined, a rearrangement of mitochondrial localization was observed, with mitochondrial clustering with higher fluorescence of the MitoTracker stain (Figure 2B,D,F). These results may be attributed to the induction of mitochondrial fusion and fission, as well as changes in mitochondrial membrane potential.

**Figure 3:**
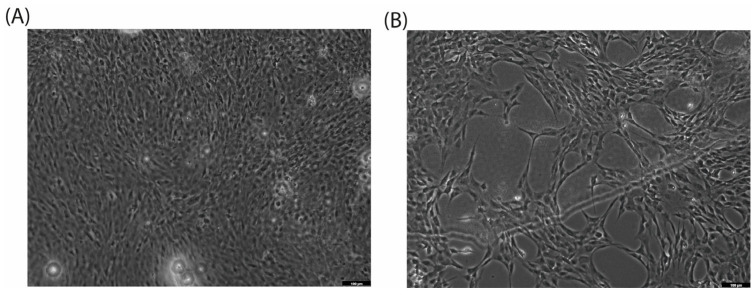
Cell differentiation after 72 h of incubation with CA (200 µg/mL, (**B**)) versus control (**A**) on C2C12 cell line. Magnification: ×10.

When cultured with CA (200 µg/mL), the C2C12 cell line exhibits a morphological switch specific for differentiation into myotubules [26]: cells elongate and form branches filled with mitochondria, and nuclei align in a line (Figure 2C,D). C2C12 were used as rapidly differentiated cells, forming contractile myotubes and producing characteristic muscle proteins. The same signs of differentiation through the myoblastic pathway under the action of CA were observed by microscopy (Figure 3).

### 2.3. Cell Death Kinetics

**Figure 4:**
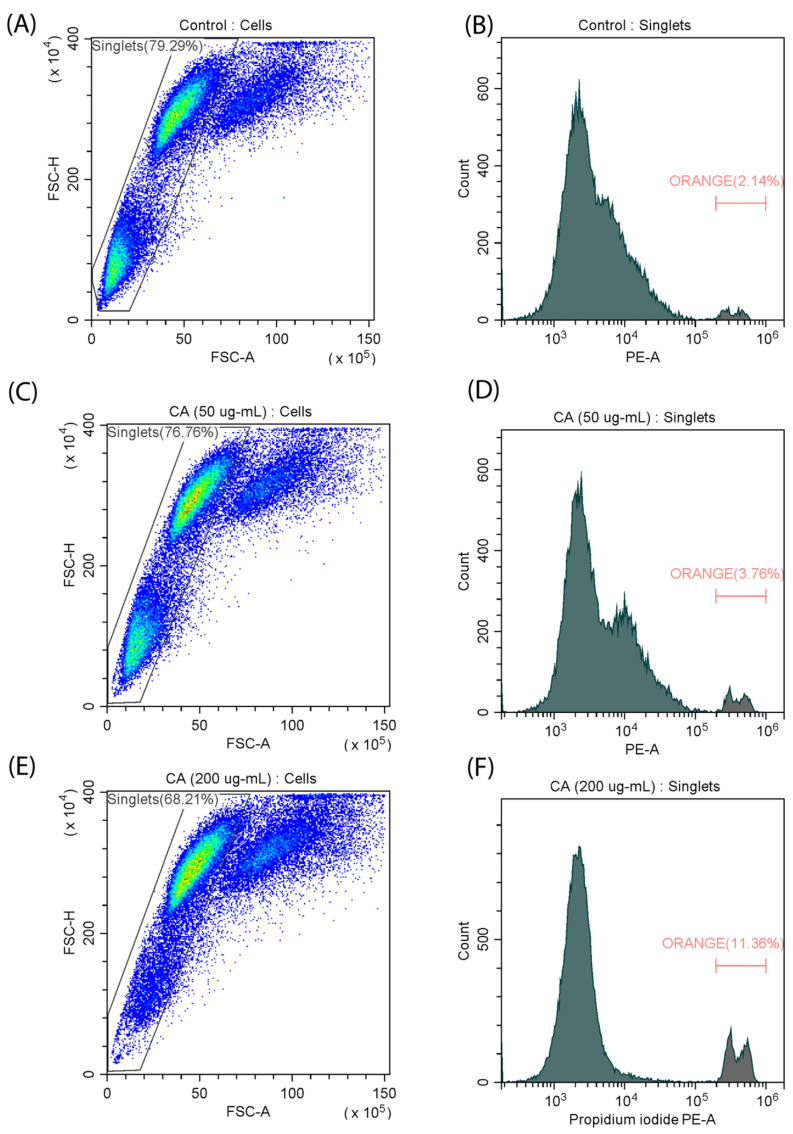
Measurement of the number of dead cells in a myoblast (C2C12) cell line stained with PI. (**A**,**B**) control samples; (**C**,**D**) incubation with CA (50 µg/mL); (**E**,**F**) incubation with CA (200 µg/mL).

To further characterize the effect of CA on cell differentiation, we decided to evaluate the cell death kinetics of the C2C12 cell line in the presence and absence of the polysaccharide. Flow cytometry analysis using the fluorescent propidium iodide (PI) dye revealed that exposure to CA at the highest concentration (200 µg/mL) resulted in a notable increase in the percentage of dead cells from 2.14% to 11.36% (Figure 4), which is consistent with the results of the MTT assay. In contrast, no significant increase in the number of dead cells was observed when a medium concentration (50 µg/mL) was used. These results suggest that cytotoxicity increases with increasing CA concentration. At the same time, an increase in apoptosis can be a characteristic of the differentiation process [27].

### 2.4. ROS Detection

**Figure 5:**
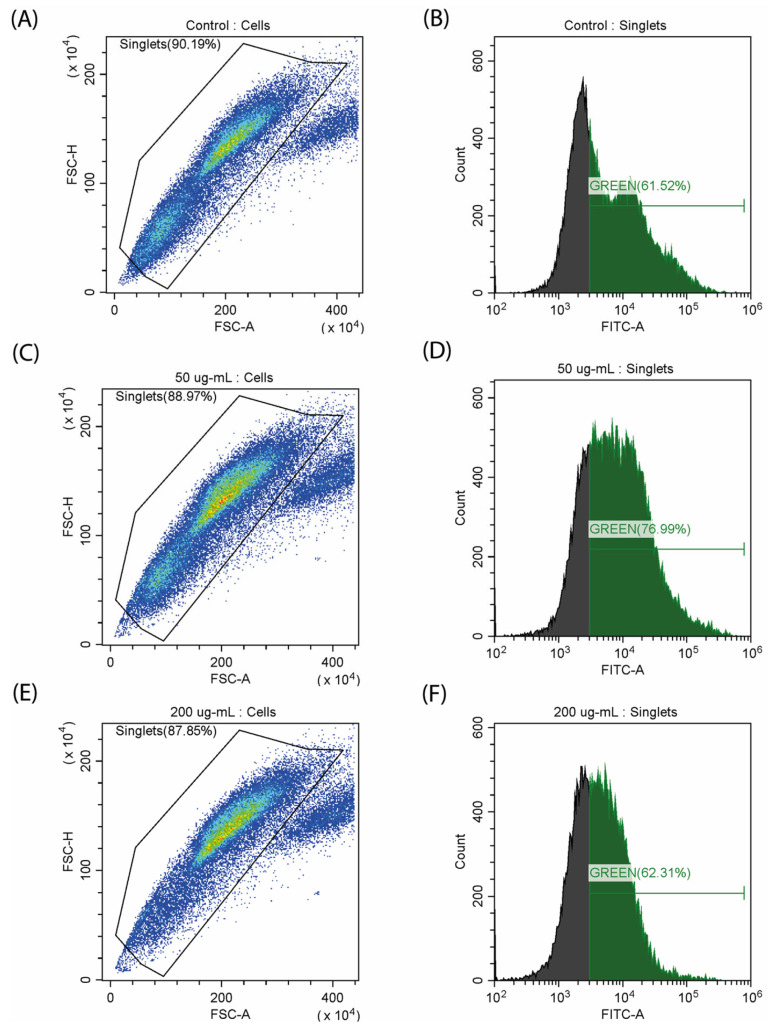
Study of ROS generation on a myoblast (C2C12) stained with carboxy-H2DCFDA. (**A**,**B**) control samples; (**C**,**D**) incubation with CA (50 µg/mL); (**E**,**F**) incubation with CA (200 µg/mL).

Characteristic features of myoblast differentiation are changes in mitochondrial activity, which is reflected in the level of reactive oxygen species (ROS) and mitochondrial membrane potential [26]. We decided to evaluate how CA affects these parameters. Quantitative staining of ROS was performed using fluorescent carboxy-H2DCFDA dye. It was observed that as the dose increased to 50 µg/mL, the amount of ROS also increased from 61.52% to 76.99% (Figure 5). The use of 200 µg/mL of CA leads to a decrease in the total number of cells and a corresponding decrease in the concentration of ROS. An increase in ROS production can be observed during C2C12 differentiation [28], and the decrease in the amount of ROS at the maximum concentration of CA can be caused by its antioxidant properties, which has been previously described for other fucose-rich polysaccharides [29].

### 2.5. Analysis of Mitochondrial Membrane Potential

**Figure 6:**
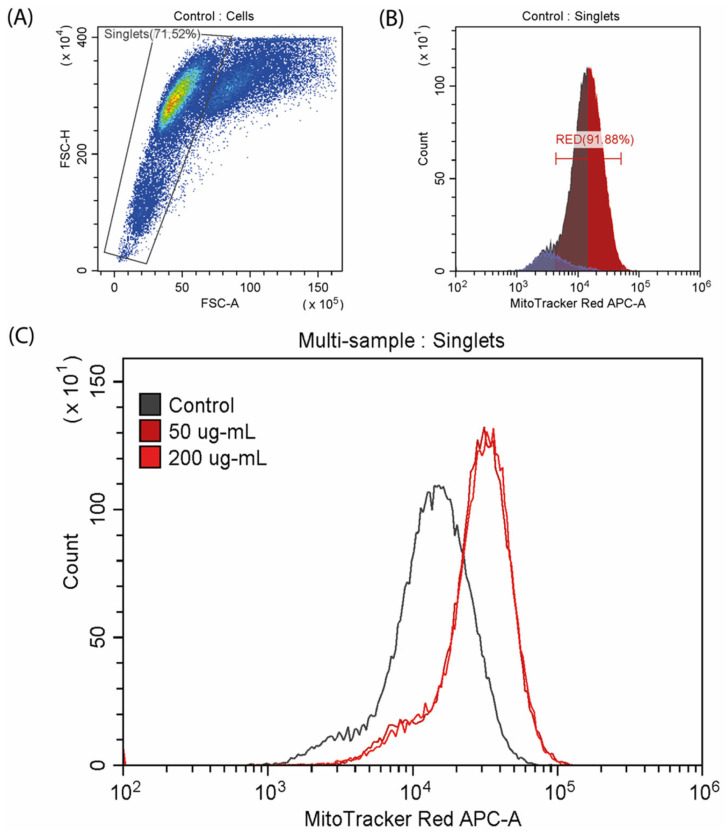
Analysis of mitochondrial membrane potential stained with MitoTracker™ Red CMXRos. (**A**,**B**) Control samples; (**C**) multi-sample analysis of mitochondrial potential in the control and CA-exposed cells.

Mitochondrial potential was investigated by flow cytometry analysis using the fluorescent MitoTracker Red CMXRos dye (Figure 6). Exposure to CA resulted in an increase in the mitochondrial potential, as indicated by a right shift of the peak. Specifically, the fluorescence intensity from the dye increased in the experimental samples treated with concentrations of 50 and 200 µg/mL, with comparable levels detected between the two concentrations.

## 3. Discussion

Bacterial polysaccharides have a wide range of biological activities, including antiproliferative, antioxidant, and antimicrobial therapy, as well as high biocompatibility and the ability to be produced on a large scale. This makes polysaccharides a promising source of new therapeutic agents. One such polysaccharide is CA, which is known for its ability to prolong the lifespans of nematodes and fruit flies [19] and to stimulate physical activity in mice [22]. In the current work, we provide evidence for the ability of CA to inhibit cell proliferation in the HCT-116 and IMR-32 cell lines as well as to induce differentiation in the C2C12 cell line and protect it from the associated ROS generation.

An analysis of the cytotoxicity of CA in the five cell lines showed that two cell lines were not affected (Vero, HPF), while the MTT assay showed that tumor-derived cell lines (HCT-116, IMR-32) experienced a decrease in their metabolic activity to about 80%. This suggests that CA exhibits potential antiproliferative activity, which has also been observed in other polysaccharides [30]. It has been previously shown that CA can influence tumor formation in *C. elegans* [19], which is consistent with the data obtained in this work. Additionally, we analyzed cell morphology and observed mitochondrial rearrangement. This is consistent with the literature describing the effect of CA on mitochondrial dynamics in the mouse fibroblast cell line (NIH/3T3) and UPRmt [19] and can be observed in different cellular processes, such as apoptosis and differentiation.

The decrease in the metabolic activity of C2C12 in the presence of CA (256 µg/mL) can be a sign of differentiation, which was confirmed in a morphology analysis. To further characterize the effect of CA on the C2C12 myoblast cell line, additional experiments were conducted. Cytotoxicity and an associated increase in the amount of apoptotic cells (from 2.14% to 11.36%) were observed in flow cytometry with PI staining. In addition, we evaluated the number of metabolically active mitochondria and observed an increase in mitochondrial membrane potential. An analysis of the ROS level showed that it increased in the presence of CA at a 50 µg/mL concentration, but higher concentrations (200 µg/mL) diminished this effect. We suggest that a decrease in ROS level at higher concentrations can be caused by the antioxidant properties of CA, which was previously shown to be dependent on the fucose content in its polysaccharide structure [29,31]. The set of observations on the changes in mitochondrial arrangement and their functional features during CA-induced differentiation complements the previously described effects of CA on UPRmt [19,32].

The results obtained need to be correlated with those published earlier. In the work by Han et al. [19], it was shown that CA can increase the lifespan of nematodes. In our work, we observed CA’s ability to stimulate differentiation of the C2C12 cell line. This is not an indicator of longevity but rather the potential for muscle regeneration, which decreases during aging [33,34]. This suggests that one of the mechanisms of the geroprotective action of CA is its ability to stimulate differentiation in myoblasts, and this effect is associated with the regulation of dynamics and metabolic activity of mitochondria.

Consequently, in our work, we confirmed previous observations that CA affects mitochondrial dynamics [19]. Here, we provide new evidence for the specificity of CA’s effects on cells of different origins, including its ability to induce differentiation and potential antiproliferative and antioxidant properties. For further investigation, CA recognition and response need to be identified.

## 4. Materials and Methods

**CA extraction and purification**. CA was produced in *Escherichia coli* S17-3 pBhya-CAB (kindly provided by Dr. Sun Junsong; Lab of Biorefinery, Shanghai Advanced Research Institute, Chinese Academy of Sciences) [35], according to a previously described protocol [22]. After incubation with antibiotics, bacterial cells were removed by centrifugation (10.500× *g*, 15 min). The supernatant was concentrated in vacuo and dialyzed against Milli-Q water for 24 h using a 12–14 kDa MWCO membrane. Trichloroacetic acid (TCA) was then added to precipitate proteins (20% *w*/*v*, 30 min), and the precipitate was separated by centrifugation (10.500× *g*, 15 min). The supernatant was collected, and 3 volumes of ethanol (95%) were added (4 °C, 24 h). The white precipitate was collected by centrifugation (10.500× *g*, 30 min), diluted in Milli-Q water, and dialyzed for 48 h. The resulting CA was lyophilized and used for further study.

**Cell lines**. Four cell lines (HCT-116, IMR-32, HPF, Vero) were provided by the N.N. Blokhin Russian Cancer Research Center, and one cell line (C2C12) was provided by the Institute of Cytology of the Russian Academy of Sciences. Four cell lines (HCT-116, IMR-32, HPF, C2C12) were grown in DMEM media (Biolot, Saint Petersburg, Russia) supplemented with 10% fetal bovine serum (Gibco, Waltham, MA, USA), 1% L-glutamine, and 1% penicillin-streptomycin at 37 °C, 5% CO_2_. The Vero cell line was incubated in a-MEM with 10% fetal bovine serum (Gibco, Waltham, MA, USA), 1% L-glutamine, and 1% penicillin-streptomycin at 37 °C, 5% CO_2_.

**MTT assay**. The MTT colorimetric assay was conducted on a 96-well plate. In total, 100 μL of cell suspension was added to each well of the 96-well microtiter plate (5000 cells/well). After 24 h of incubation, the CA was diluted in a growth medium and added in concentrations from 2 to 256 μg/mL. The plates were then incubated (37 °C, 72 h, 5% CO_2_). After incubation, cultures were incubated with MTT (2 h) and washed with DMSO. Optical density of formazan was measured at 570 nm on a Tecan Infinite microplate reader (Switzerland). All experiments were conducted in a triplicate, and mean values with standard deviations were calculated.

**Fluorescence microscopy**. Cells were seeded on cover glasses (d 11 mm, Thermo FS, Waltham, MA, USA) in a 24-well plate (10 × 10^3^ cells per well) in DMEM media (Biolot, Russia) supplemented with 10% fetal bovine serum (Gibco, Waltham, MA, USA), 1% L-glutamine, and 1% penicillin–streptomycin at 37 °C, 5% CO_2_ for 24 h. Then, growth medium was replaced with CA solution (200 ug/mL in growth medium) and incubated for 72 h. After that, cells were stained with MitoTracker™ Red CMXRos (Thermo FS, Waltham, MA, USA) for 30 min. Then, the cells were washed 3 times with phosphate-buffered saline (PBS) to remove any free dye. Cells were fixed with 4% paraformaldehyde (PFA) solution for 15 min and stained with DAPI solution (Thermo FS, Waltham, MA, USA) for 5 min. Samples were replaced on slide glass in glycerin and fixed with lacquer. Cell morphology was evaluated with a Leica DMi8 fluorescence microscope equipped with a Leica DFC365FX digital camera (Leica, Wetzlar, Germany).

**Analysis of apoptosis in C2C12 cells**. The kinetics of cell death were evaluated by propidium iodide (PI). The cells were seeded on cover glasses, as described above. After 24 h of incubation, the CA was added in concentrations of 50 and 200 μg/mL, and the culture was incubated for 72 h. Cells were transferred into tubes and resuspended in PBS. After that, cells were stained in DMEM with PI (10 µg/mL) for 2 min in the dark. Fluorescence intensity was measured in the phycoerythrin channel on CytoFlex (Beckman Coulter, Brea, CA, USA). Data were analyzed using CytExpert (Beckman Coulter, Brea, CA, USA) and Microsoft Excel 2016.

**ROS detection in C2C12 cells**. CM-H2DCFDA dye (Thermo Fisher, Waltham, MA, USA) was used to detect ROS and general oxidation stress [36]. The cells were seeded on cover glasses, as described above. After 24 h of incubation, the CA was added in concentrations of 50 and 200 μg/mL, and the culture was incubated for 72 h. Cells were transferred into tubes and resuspended in PBS. Then, they were treated with 5 µM of dye for one hour. The intensity of fluorescence was measured in the FITC channel on CytoFlex (Beckman Coulter, Brea, CA, USA). Data were analyzed using CytExpert (Beckman Coulter, Brea, CA, USA) and Microsoft Excel 2016.

**Mitochondrial membrane potential in the C2C12 cells**. The cells were seeded on cover glasses, as described above. After 24 h of incubation, CA was added in concentrations of 50 and 200 μg/mL, and the resulting culture was incubated for 72 h. After that, the cells were stained in DMEM using 150 μM MitoTracker™ Red CMXRos (Thermo FS, Waltham, MA, USA) for 30 min. Cells were transferred into tubes and resuspended in PBS. Next, samples were incubated with 85 nM of MitoTracker in PBS for 30 min. Fluorescence intensity was measured on CytoFlex (Beckman Coulter, Brea, CA, USA). Data were analyzed using CytExpert (Beckman Coulter, Brea, CA, USA) and Microsoft Excel.

## 5. Conclusions

In conclusion, our study aimed to describe the cellular effects caused by the action of the bacterial exopolysaccharide CA. We observed that CA acts specifically in cell lines of different origins, possibly affecting processes such as mitochondrial dynamics, proliferation, and differentiation. A standard model for studying differentiation, the C2C12 cell line, showed that CA can induce differentiation, which was confirmed by changes in cell morphology, apoptosis, ROS generation, and an increase in mitochondrial membrane potential. Thus, we confirm that CA affects mitochondria and propose a new potential use of CA as a component of cancer therapy and an antioxidant. However, further investigations are required to describe the mechanisms of CA’s influence on eukaryotic cell physiology.

## Data Availability

Data are contained within the article.
